# Quantifying the Financial Return on Investment of Risk-Based Quality Management Implementation in Clinical Development

**DOI:** 10.1007/s43441-026-01002-1

**Published:** 2026-06-25

**Authors:** Abigail Dirks, Sylviane de Viron, Kenneth McFarlane, Kenneth A. Getz

**Affiliations:** 1 Tufts Center for the Study of Drug Development, Tufts University School of Medicine, Boston, MA USA; 2 Clue Points S.A, Louvain-la-Neuve, Belgium; 3 CluePoints Inc, King of Prussia, PA USA

**Keywords:** Risk-based quality management, Quality by design, Centralized monitoring, Source data verification, Expected net present value, Return on investment, Clinical trial efficiency, Oncology drug development, ICH E6(R3)

## Abstract

**Background:**

Despite widespread receptivity to risk-based quality management (RBQM) principles, adoption has been limited as drug development stakeholders have lacked quantitative evidence demonstrating return on investment (ROI).

**Methods:**

Two assessments were conducted to estimate the ROI of RBQM. (1) Clinical trial level ROI was derived by analyzing monitoring cost efficiencies and clinical phase duration reductions under a 10% source data verification (SDV) scenario. (2) Program-level ROI was derived using an expected net present value (eNPV) modeling approach. Both analyses included data based on actual RBQM use in 18 recently completed oncology trials, as well as published and proprietary industry benchmarks.

**Results:**

RBQM use was associated with reductions of 8–19% in clinical phase durations and monitoring cost reductions of up to 18% under a 10% SDV scenario. Total clinical trial ROIs ranged from $3.2 million (phase 1) to $18.9 million (phase 3), corresponding to ROI multiples of 6× to 23×. Program-level eNPV gains ranged from $3.8 million in phase 1 to $13.8 million in phase 3 corresponding to ROI multiples of 4× to 14×.

**Conclusions:**

The ROI demonstrated at the individual clinical trial level and at the development program level provides a compelling business case for adopting RBQM particularly given alignment with ICH E6 R3 guidelines encouraging risk-based approaches supporting clinical trial planning and execution. Study findings indicate that RBQM-supported oversight not only improves data quality and risk detection but also represents a strategic investment that can help optimize resource allocation, increase development efficiency and speed, and enhance portfolio productivity.

## Introduction

Risk-based quality management (RBQM) has emerged as a critical component of modern clinical trial oversight, reflecting a broad shift toward proactive, data-driven approaches to ensure patient safety, protocol compliance, and data integrity. Grounded in the principles of Quality by Design (QbD), RBQM encourages sponsors to identify, evaluate, and manage critical risks early and continuously throughout clinical trial execution [1, 2]. As clinical trials have grown in scope and complexity—driven by increasing data volume, decentralization, and operational variability—RBQM has become increasingly important for maintaining high-quality study conduct in a resource-efficient manner [1–5].

RBQM is typically operationalized through several core components: prospective risk assessment, identification of critical-to-quality (CtQ) factors, centralized and statistical data monitoring (SDM), the use of key risk indicators (KRIs) and quality tolerance limits (QTLs), and targeted source data verification (SDV). Together, these practices shift oversight from routine, visit-based monitoring toward continuous, remote and data-informed surveillance, enabling sponsors to detect emerging risks earlier and directing monitoring resources more efficiently [1, 2, 4, 5].

Regulatory agencies have long recognized the need for more risk-proportionate monitoring and oversight. Initial guidance from the U.S. Food and Drug Administration and the European Medicines Agency introduced RBQM concepts more than a decade ago [1, 2] and the release of ICH E6(R3) in 2025 marks a significant milestone in modernizing Good Clinical Practice and encourages sponsors to adopt risk-based approaches to monitoring and oversight [5].

Despite regulatory support and the operational benefits reported anecdotally and in case studies, RBQM adoption across the industry has been slow and inconsistent. Large pharmaceutical companies report that they have implemented RBQM components on an average of 63% of active clinical trials in their portfolios each year, while smaller companies report that they implemented RBQM components on less than half (48%) of their active clinical trials annually [6].

Sponsors cite barriers including limited cross-functional awareness and support; insufficient operational readiness, and inadequate change-management infrastructure [6].

A study conducted by the Tufts Center for the Study of Drug Development (Tufts CSDD) in 2022 corroborates these findings and suggests that the demonstration of a quantifiable return on investment (ROI) and clear regulatory agency support are two essential factors most contributing to organizational commitment to fully implement innovations supporting clinical research operating activity [7].

Studies in the literature show that RBQM enables earlier detection of emerging risks—such as inadequate adverse-event reporting, protocol deviations, or delays in data entry—while reducing the need for routine on-site monitoring and improving the allocation of monitoring resources. These risks can be identified at multiple levels (e.g., site, country, or region), providing a structured, data-driven framework for detecting anomalies and proactively remediating data quality concerns [8–11]. However, these findings remain largely descriptive and do not quantify the financial value of RBQM. To our knowledge, there is no research that has systematically quantified the economic return on RBQM investment using rigorous financial modeling based on actual implementation experience. This limits confidence among sponsors considering beginning and/or scaling portfolio-wide adoption of RBQM practices.

This study addresses this evidence gap by quantifying the financial value of RBQM-supported oversight at both the clinical trial and development program levels. The objective of this research was to evaluate how RBQM influences direct study costs, time efficiencies, and overall economic value, providing a data-driven foundation to support broader RBQM adoption.

## Methods

### Data Selection

Data for this analysis were drawn from three primary sources: published benchmarks, internal clinical trial datasets, and real-world RBQM implementation data.

Published benchmarks provided information on clinical trial costs, monitoring practices, phase durations, commercial parameters, and industry-standard assumptions for site monitoring and SDV, with oncology-specific values applied where available [12–14]. These data were used to establish baseline (non-RBQM) values for comparison (Table 1). Non–phase-specific inputs were incorporated directly into the model, including monitoring expenditures assumed at 12% of total R&D cost, on-site visit costs of $3600 plus $800 for travel, and a visit frequency of every 3 weeks [15]. Baseline SDV level was fixed at 100%, and assumptions for the average number of pre-approval (3.14) and post-approval (5.4) indications studied and approved per oncology drug were based on published oncology benchmarks [14].

**Table 1:** Baseline parameters for an oncology development program

Parameter	Phase 1	Phase 2	Phase 3
Number of trials per program [12]	2.0	2.6	1.2
Mean duration to primary completion (days)	1021.6	1262.8	1308.4
Contribution to total program duration (%)	34.3	24.0	35.0
Transition success rate [13] (%)	48.4	24.6	47.7
R&D cost per trial per phase	$14.2 M	$25.5 M	$65.8 M

Values reflect oncology-specific benchmark data used to populate the base-case and RBQM-case models. All costs are inflation-adjusted to current-year dollars. Durations are measured from study initiation to primary completion. Transition success rates represent the likelihood of progressing to the next phase

**Fig. 1:**
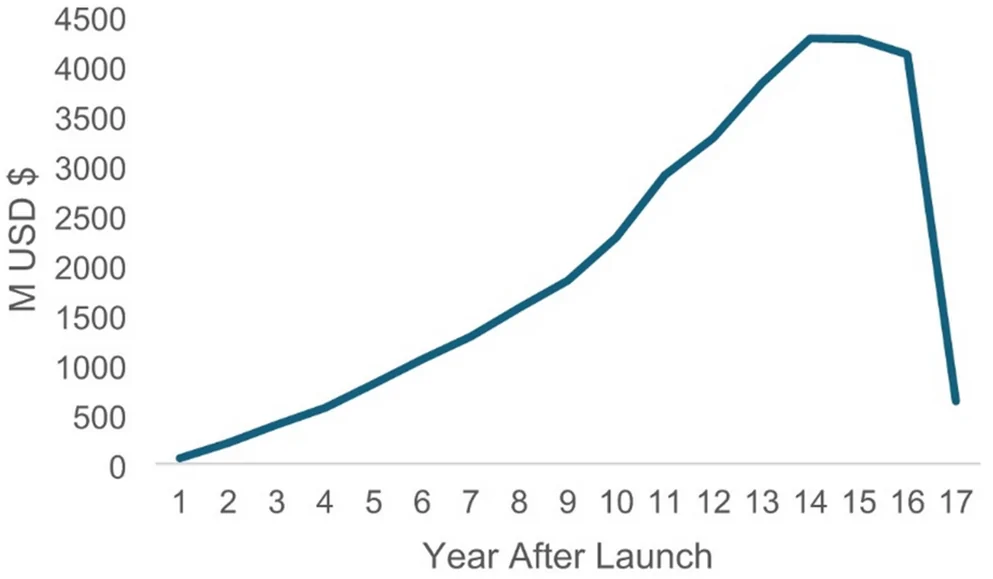
Mean revenue trajectory for oncology drugs (Inflation-Adjusted to 2014 Launch)

Tufts CSDD internal datasets contributed detailed cost information from oncology clinical trials, including study-level budgets, historical cycle times, and monitoring-resource utilization. These datasets comprise comprehensive study budgets from 409 protocols with primary completion or database lock dates between 2016 and 2021. They include standardized cost structures and daily cost estimates that were used to calculate inflation-adjusted, present day valuations of clinical trial time (Fig. 1) [16].

Real-world RBQM usage data were obtained from 18 oncology clinical trials managed through the CluePoints RBQM platform. The dataset represented a diverse operational sample across seven sponsors, including both pharmaceutical companies and contract research organizations. Most trials were multi-country studies (83%), and the sample covered multiple oncology disease categories, including urogenital, skin and connective tissue, hemic and lymphatic, digestive system, nervous system, and respiratory tract malignancies. These data reflect a minimum level of RBQM deployment, consisting of the use of centralized statistical monitoring and the application of KRIs within the platform. Sponsors may have used additional RBQM components—such as QTLs, targeted SDV, enhanced risk assessment procedures, or other operational tools—either within or outside the CluePoints environment. The dataset therefore captures a consistent baseline of RBQM activity, while acknowledging variability in the broader RBQM strategies applied across studies. Because the 18 oncology trials did not include complete monitoring-cost details, hypothetical values for monitoring visit frequency, on-site and remote monitoring costs, and RBQM technology and service investment costs were derived using CluePoints RBQM operational guidelines and oncology-aligned monitoring cost frameworks. These harmonized assumptions ensured consistency across analyses and allowed for direct comparison between the baseline and RBQM scenarios.

All data inputs were reviewed for completeness and adjusted to present day dollar values using inflation indices. The final dataset represents an integrated foundation combining oncology-specific empirical RBQM usage data with benchmarked and guideline-based cost and monitoring assumptions appropriate for modeling the financial impact of RBQM implementation.

### Statistical Analysis

#### Clinical Trial ROI Analysis

The objective of this assessment was to quantify the direct financial impact of RBQM implementation in individual clinical trials. A cost model was developed to estimate two primary components of financial value: (1) Monitoring cost efficiencies, including reductions in on-site monitoring visits and travel costs associated with lower SDV requirements; and (2) The financial value of time saved, reflecting shorter clinical phase durations observed in RBQM-enabled trials.

Monitoring cost savings were calculated by comparing the total cost of monitoring activities under a baseline assumption of 100% SDV with the RBQM scenario, which applied empirically observed lower SDV rates and corresponding reductions in on-site monitoring frequency. Cost elements included on-site visit labor, travel, and remote follow-up costs.

Time savings were estimated by applying observed percentage reductions in phase durations to benchmark timelines. The financial value of these time savings was derived using standardized estimates of daily clinical trial cost, adjusted to current dollars. Total financial benefit at the clinical trial level was calculated as the sum of monitoring cost savings and the financial value of time. ROI was computed as the ratio of total financial benefit of RBQM to the deployment investment required.

#### Program-Level ROI Analysis

The second assessment quantified the net financial value of RBQM across a full clinical development program using an eNPV modeling framework. This widely accepted modeling approach incorporates key elements of development investment and value creation, including research and development costs, phase durations, transition probabilities, revenue forecasts, patent or exclusivity periods, and the cost of capital [13–15].

For each clinical phase and phase combination, two scenarios were modeled: (1) A base case, assuming conventional development practices without RBQM and 100% SDV; and (2) An RBQM case, applying observed reductions in phase duration and monitoring costs from RBQM-enabled trials.

All cash flows were normalized to the start of the modeled phase. eNPV values were computed by discounting future net cash flows using a standard weighted average cost of capital. The net financial value created by RBQM was defined as the difference (delta) between the eNPV of the RBQM case and the eNPV of the base case (without RBQM). ROI multiples were derived by dividing eNPV delta by the RBQM technology and service investment cost.

### Sensitivity Analysis

Sensitivity analyses were performed to evaluate the robustness of model outputs under different assumptions. Key parameters—specifically, the magnitude of phase-level time savings and the cost of RBQM implementation—were varied by ± 25% to measure changes in ROI based on varying levels of investment and time savings. Each scenario was recalculated to assess the effect of these changes on the eNPV delta and ROI. Break-even thresholds were examined to identify conditions under which RBQM would cease to generate net positive financial value.

## Results

### Clinical Trial ROI Analysis

RBQM-enabled trials were associated with measurable financial efficiencies across all clinical trial phases. Under the 10% SDV scenario, monitoring cost savings reduced total monitoring expenditures by up to 18% compared with the baseline assumption of 100% SDV (Table 2).

**Table 2:** Monitoring cost reduction by source data verification scenario

% SDV (%)	Phase 1 (%)	Phase 2 (%)	Phase 3 (%)
10	− 18	− 9	− 11
20	− 16	− 8	− 9
50	− 10	− 5	− 6
75	− 5	− 2	− 3

Values represent percentage reductions in total monitoring costs relative to a baseline assumption of 100% SDV. Monitoring-cost assumptions include an estimated on-site visit cost of $3600, travel cost of $800 per visit, and a visit frequency of every 3 weeks

**Fig. 2:**
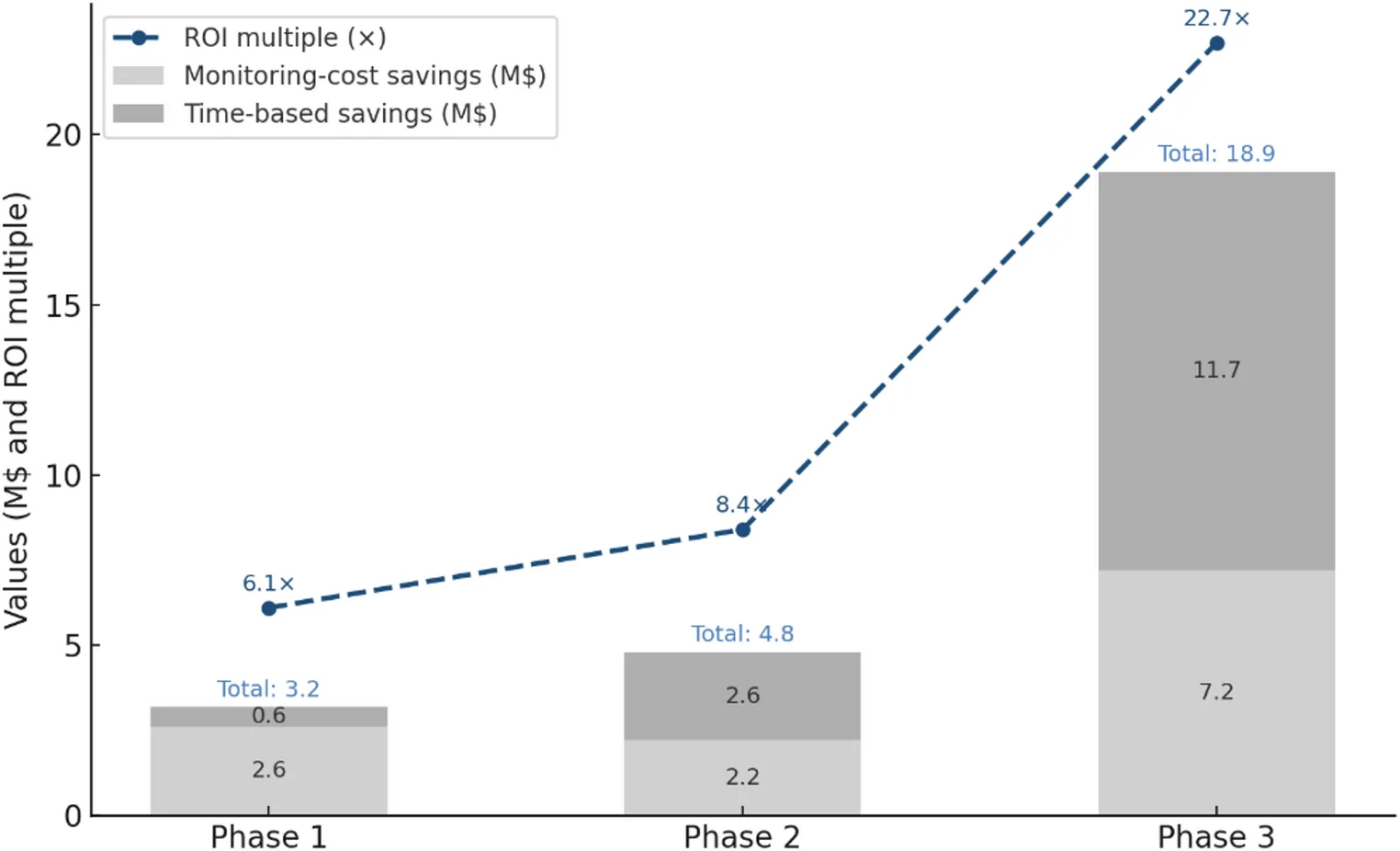
Clinical trial level ROI assessment of RBQM implementations at 10% SDV. Monitoring cost efficiency savings and time-based financial savings are shown as stacked bars. ROI multiples are displayed as a dashed line on the same scale. All values are derived under a scenario in which 10% of eCRF source data are reviewed and verified (SDV), compared with a baseline of 100% SDV

Reductions in clinical trial durations ranged from 8% in phase 1 to 19% in phase 3 (Table 3), generating time-based financial savings of $0.6 million, $2.6 million, and $11.7 million in phases 1, 2, and 3, respectively (Fig. 2).

**Table 3:** Phase duration and time reduction

Phase	% of total development time	% Time saved (RBQM)
Phase 1	20.1	− 8.3
Phase 2	23.2	− 9.6
Phase 3	34.9	− 18.6

Percent of total development time reflects the contribution of each phase to the overall oncology program duration. Percent time saved represents reductions in phase duration observed in RBQM-enabled trials compared with the baseline case

The clinical trial-level benefit of RBQM deployed by phase—combining monitoring-cost efficiencies and the financial value of time saved—is summarized in Fig. 2, with a returns of $3.2 million in phase 1, $4.8 million in phase 2, and $18.9 million in phase 3. Corresponding ROI multiples by phase were 6.1×, 8.4×, and 22.7×, respectively.

### Program-Level ROI Analysis

The analysis suggested that RBQM generated positive financial value at the development program level across all scenarios modeled. For single-phase implementations, eNPV deltas were $3.76 million in phase 1, $2.75 million in phase 2, and $13.83 million in phase 3. For combined-phase scenarios, eNPV deltas were also positive, ranging from $1.31 million for phases 1–3 combined to $3.71 million for phases 2–3. Corresponding ROI multiples ranged from 3.9× to 13.5×, with the highest values observed in phase 3. Across all scenarios, time savings accounted for the majority of financial value created, representing 75–97% of the total eNPV delta (Table 4).

**Table 4:** Program level ROI assessment of RBQM implementation (eNPV modeling)

Scenario	eNPV base case (M$)	eNPV RBQM case (M$)	eNPV delta (M$)	ROI multiple	% Due to cost savings	% Due to time savings
Phase 1	180.10	183.86	3.76	3.9×	3.4	96.6
Phase 2	84.23	86.99	2.75	4.1×	19.4	80.6
Phase 3	169.92	183.76	13.83	13.5×	16.3	83.7
Phases 1–2	38.43	39.96	1.53	3.9×	14.6	85.4
Phases 2–3	34.12	37.83	3.71	9.1×	3.8	96.2
Phases 1–2–3	13.61	14.92	1.31	4.6×	25.2	74.8

All eNPV values are discounted to the start of the earliest modeled phase. Results assume a 10% SDV scenario for RBQM and a baseline of 100% SDV. ROI multiples are calculated as the ratio of eNPV delta to the RBQM investment cost

### Sensitivity Analysis

When time-savings assumptions were reduced by 25%, eNPV deltas remained positive in all but the phase 2 scenario, which showed a slightly negative value when combined with a 25% increase in RBQM investment. Increasing time-savings assumptions by 25% raised eNPV deltas across all scenarios. Changes in RBQM investment costs had a smaller effect on results than changes in time savings. RBQM deployments in Phase 3 were associated with positive net financial value under all sensitivity conditions (Tables 5 and 6).

**Table 5:** Phase 2 sensitivity analysis results (Millions USD)

Time savings assumption	Investment − 25%	Baseline investment	Investment + 25%
− 25%	0.77	− 0.60	− 1.06
Baseline	4.05	2.75	2.32
+ 25%	4.88	3.61	3.19

Values represent eNPV deltas (in millions USD) under variations of ± 25% in time-savings assumptions and RBQM investment costs. All results assume a 10% SDV scenario for RBQM and a baseline of 100% SDV for the conventional case

**Table 6:** Phase 3 sensitivity analysis results (Millions USD)

Time savings assumption	Investment − 25%	Baseline investment	Investment + 25%
− 25%	3.30	2.31	3.30
Baseline	14.69	13.83	13.55
+ 25%	18.08	17.23	16.99

Values represent eNPV deltas (in millions USD) under variations of ± 25% in time-savings assumptions and RBQM investment costs. Phase 3 remained value-generating under all tested conditions. Analyses assume a 10% SDV scenario for RBQM and a baseline of 100% SDV

## Discussion

Pharmaceutical and biotechnology companies have shown reluctance to adopt RBQM due to insufficient evidence quantifying its value proposition. This study addresses that gap by estimating the ROI and net financial value associated with RBQM-supported oversight at both the clinical trial and development program levels.

At the clinical trial level, RBQM-enabled oncology trials were associated with measurable monitoring-cost efficiencies and duration reductions. Across the 18 trials analyzed, clinical phase durations were reduced by 8% in phase 1 to 19% in phase 3, and 10% SDV of eCRF data yielded study monitoring cost efficiencies of as much as 18% compared to industry benchmarks. The results of the clinical trial ROI assessment showed a positive return of between 6 and 23 times the investment in direct monitoring cost and time savings to implement RBQM on a single oncology clinical trial by phase. Whereas most trial-level financial benefit in shorter-duration phase 1 trials was associated with monitoring efficiency, in longer-duration phase 2 and phase 3 trials a larger share of the total financial benefit was attributable to time efficiencies.

At the program level, positive eNPV deltas were observed for each phase and phase combination. The net financial value associated with an RBQM-supported program—compared to a typical oncology drug development program that did not have the benefit of RBQM—ranged from $2.8 million in phase 2 implementations to $13.8 million in phase 3 implementations representing multiples of 4–14 times the investment. RBQM implementations across multiple phase combinations also yielded positive eNPV deltas ranging from $1.3 million to $3.7 million. The primary driver of net financial value in the eNPV model was time savings associated with more focused monitoring activities, earlier identification of operational and data-quality risks, and reduced delays in issue resolution, study close-out, and completion. Although QbD and RBQM planning activities may require additional effort at study start-up, these activities can support better prioritization of critical risks and more efficient downstream execution. Sensitivity analyses showed that eNPV deltas remained positive across most scenarios. The main exception was phase 2, where the comparatively lower ROI reflects the risk-adjusted structure of the eNPV model rather than an absence of operational benefit. Although phase 2 RBQM-enabled trials generated monitoring and time efficiencies, the expected financial value of those efficiencies is reduced by the lower probability of progression from phase 2 through later development and approval. At the same time, because phase 2 trials are longer than phase 1 trials, phase 2 estimates were more sensitive to changes in time-reduction assumptions and RBQM investment levels. The higher ROI observed at the single-trial level compared with the development-program level is also expected because the eNPV assessment is risk-adjusted, incorporates successful and unsuccessful development outcomes, and discounts value back to the beginning of the modeled clinical phase.

These findings build on the existing RBQM literature, which has primarily focused on operational rather than financial outcomes. Prior research has demonstrated that centralized monitoring and statistical data surveillance can detect data irregularities, protocol deviations, and safety signals earlier than traditional monitoring approaches [8, 9, 17, 18]. Studies have also shown that targeted SDV, SDM, KRIs, and QTLs can improve oversight effectiveness and direct monitoring resources more efficiently [8, 9, 17, 18]. The present study extends this literature by quantifying the financial magnitude associated with monitoring efficiencies and time savings, thereby addressing a longstanding gap in the evidence base for RBQM adoption.

The results also have important implications for industry adoption. Sponsor companies have frequently cited uncertainty about the financial return on RBQM investments as a primary barrier to broader adoption, even as regulatory expectations for risk-based oversight continue to intensify [19]. The ROI associated with RBQM-supported oversight at both the individual clinical trial and development program levels provides a business case for increased adoption. These findings support the view that RBQM-supported approaches can help optimize monitoring resources, reduce development timelines, and enhance portfolio productivity while aligning with evolving expectations under ICH E6(R3).

There are a number of limitations to note in our assessments that both suggest future research opportunities and indicate that our findings are conservative. Our assessments only looked at oncology development programs and used benchmark averages to derive estimates and to populate the eNPV model. The impact of RBQM implementations will no doubt vary considerably by therapeutic area and by the size of the patient community living with a particular unmet medical need. Future analyses will gather benchmarks on other therapeutic areas and patient communities. Although it is possible that the benchmark sample of protocols may have been influenced by the deployment of select RBQM components, the likelihood is very low based on published historical adoption levels. The benchmark sample is drawn from protocols that had primary completion dates no later than 2015 when adoption of RBQM components was nascent. The comparison group of protocols, however, definitively benefited by the comprehensive deployment of RBQM components.

The ROI and net financial value estimates derived in this study only partially measure total investment and total financial impact. As noted earlier, the financial investment is based solely on the cost to implement RBQM technology and services. It does not include recurring operational costs such as training, data integration, central monitoring resourcing, governance or vendor oversight. It also does not include one-time implementation costs such as new operating procedures, maintenance of legacy monitoring processes, and change management activities. Conversely, the analysis did not quantify financial value associated with earlier detection of data discrepancies, anomalies, or fraud; reduced rework; improved data integrity; fewer late-stage quality issues; or potential improvements in inspection readiness and technical or regulatory success. Lastly, these assessments do not include the intangible benefits of RBQM including improvement in study monitor job satisfaction, freed capital and resources to be put toward the development of new investigational treatments, and faster time to delivering a new therapy to patients and their families. These exclusions suggest that the results should be interpreted as technology- and service-based ROI rather than a comprehensive enterprise-level cost-benefit assessment. Future research should assess these broader RBQM-related value drivers directly, including intangible benefits such as study monitor job satisfaction, freed capital and resources, and faster delivery of therapies to patients.

## Conclusion

In conclusion, this study provides quantitative evidence that RBQM-supported oversight is associated with meaningful financial value at both the clinical trial and development program levels. By translating monitoring efficiencies and time savings into ROI and eNPV outcomes, the analysis helps address a key barrier to RBQM adoption: the lack of a clear financial value proposition. These findings are particularly relevant as ICH E6(R3) reinforces the expectation that sponsors use proactive, risk-based approaches to clinical trial planning, monitoring, and oversight.

## Data Availability

It is Tufts CSDD’s policy to make de-identified raw data available for scholarly analysis.
